# Dating the age of admixture via wavelet transform analysis of genome-wide data

**DOI:** 10.1186/gb-2011-12-2-r19

**Published:** 2011-02-25

**Authors:** Irina Pugach, Rostislav Matveyev, Andreas Wollstein, Manfred Kayser, Mark Stoneking

**Affiliations:** 1 Max Planck Institute for Evolutionary Anthropology, Deutscher Platz 6, Leipzig, D-04103, Germany; 2 Institute for Mathematics, University of Leipzig, PF 10 09 20, Leipzig, D-04009, Germany; 3 Cologne Center for Genomics, University of Cologne, Weyertal 115b, Cologne, D-50931, Germany; 4 Department of Forensic Molecular Biology, Erasmus MC University Medical Center Rotterdam, Postbus 2040, Rotterdam, 3000 CA, The Netherlands

## Abstract

We describe a PCA-based genome scan approach to analyze genome-wide admixture structure, and introduce wavelet transform analysis as a method for estimating the time of admixture. We test the wavelet transform method with simulations and apply it to genome-wide SNP data from eight admixed human populations. The wavelet transform method offers better resolution than existing methods for dating admixture, and can be applied to either SNP or sequence data from humans or other species.

## Background

An admixed population arises when individuals from two or more distinct populations start exchanging genetic material. Studying admixed populations can be particularly useful for understanding differences in disease prevalence and drug response among different populations. There is ample evidence that human populations have different susceptibility to diseases, exhibiting substantial variation in risk allele frequencies [1]. For example, genetic predisposition to asthma differs among the differentially-admixed Hispanic populations of the United States, with the highest prevalence observed in Puerto Ricans. Genetic variants responsible for the increased asthma prevalence in this population were localized using an admixture mapping approach [2]. This method allows the identification of disease causing variants by estimating ancestry along the genome, and narrowing the search to the genomic regions with ancestry from a population that has a greater risk for the disease [3,4]. The same approach was used to identify genetic loci that influence susceptibility to obesity, which is about 1.5-fold more prevalent in African-Americans than in European-Americans [5].

Admixed populations are also of interest to population geneticists as they offer invaluable insights into the impact of various human migrations. For example, Polynesian populations are of dual Melanesian and Austronesian ancestry, with more maternal Austronesian and paternal Melanesian ancestry, highlighting the importance of sex-specific processes in human migrations [6]. The analysis of the pattern of sharing of chromosomal regions between populations has provided important insights into human colonization history including multiple migration waves into the Americas, and a complex movement of people across Europe [7]. A study of admixture patterns in Indian populations revealed that most Indians today trace their ancestry to two ancient, genetically-divergent populations [8].

Analyses of admixture patterns in human populations have also proven useful for studies of local selection. The genomewide distribution of ancestry has been examined and signals of recent selection have been identified in admixed populations of Puerto Ricans [9] and African Americans [10].

Over the years various methods have been developed to study genetic ancestry both at the level of an entire population [11], and at the level of individuals within admixed populations [4,10,12-17]. Because genetic recombination breaks down parental genomes into segments of different sizes, the genome of a descendant of an admixture event is composed of different combinations of these ancestral segments, or 'blocks'. The distribution of ancestry proportions within a population and the structure of an admixed genome can thus provide information on the timing of the admixture event itself. Previously, a likelihood-based method (HAPMIX) was developed to infer the time of admixture events from the haplotype block information [17]. Here we introduce a PCA-based genome scan approach to detect and date admixture events. Stepwise principal component analysis is carried out along each chromosome of an admixed individual and respective parental populations, and spectral decomposition of the resulting signal is used to infer the date of admixture. We validate the method on simulated data sets, and on a sample of African Americans, as a population with known admixture history. To test how the performance of our approach compares to HAPMIX, which uses a fundamentally different methodology to infer local ancestry, we apply our method to the Human Genome Diversity Panel (HGDP) populations [18] for which European admixture has been estimated and dated using HAPMIX [17]. Finally, we apply the method to elucidate the structure and admixture proportions, and estimate admixture time, in a Fijian population and in a diverse sample of Polynesians [19].

## Results and discussion

### Overview of the method

**Figure 1:**
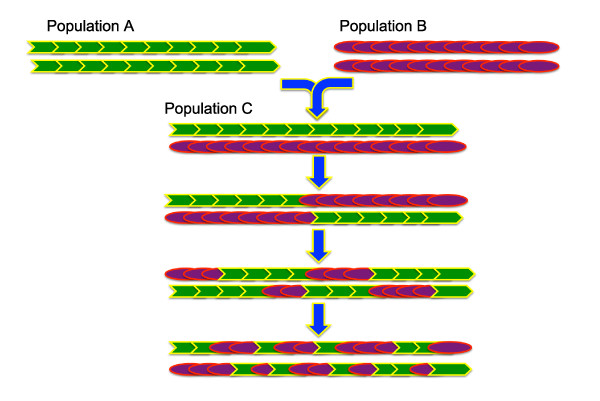
**Diagram giving an overview of the admixture process**. When two populations admix, genetic recombination starts breaking ancestral genomes into blocks of different sizes, so that the genomes of the descendants of an admixture event are composed of different combinations of these ancestral blocks. The number and width of the admixture blocks contain information about the time since admixture, as more recombination events result in a greater number of blocks, which with time get progressively narrower and more evenly spread along and among chromosomes.

The idea behind the method is straightforward: when two populations admix, genetic recombination starts breaking 'ancestral' genomes into blocks of different sizes, so that the genomes of the descendants of an admixture event are composed of different combinations of these ancestral blocks (Figure 1). Hence, by screening the genome of an individual of mixed ancestry, we identify stretches of the genome which are inherited from either of the ancestral populations. Moreover, the structure of an admixed genome contains information on the timing of the admixture event itself. The number of admixture blocks reflects past recombination events, and similarly the width of such blocks also contains temporal information, as more recombination events would result in narrower blocks that are more evenly spread along and among chromosomes.

To analyze local genomic admixture structure for an individual in an admixed population and to use such structure to infer the date of admixture we introduce a two-part method. The first part of the method, named StepPCO, is an extension of principal component analysis (PCA) and is used to obtain a signal of admixture from an individual genome. The second part of the method relies on the wavelet decomposition of this admixture signal to extract information about the date of the admixture event. Here we provide a descriptive overview of the method; the actual methodology is formally developed in the Materials and Methods section below.

We start by performing a sequential stepwise PCA (StepPCO) along each chromosome of an individual from an admixed population and of individuals from respective parental populations. We consider an admixed population as a mixture of two ancestral populations, in which the admixture occurred at a single timepoint, and assume that no genetic drift occurred after the admixture event. These, of course, are simplifying assumptions, as most human populations are expected not only to have many incidents of admixture occurring at different points in time, and between different populations, but also to experience genetic drift relative to the parental populations. We try to circumvent this issue by finding the first principal axis (PA1) based on the samples from the proposed ancestral populations or their proxies, and then project the admixed dataset onto the axis of variation defined by these ancestral populations, thereby excluding any signal which potentially could originate from drift and/or other sources of ancestry [8,20]. We then consider a sliding window along each chromosome. The size of this window is not fixed, but at each position is determined by the statistical properties of the collection of SNPs in the window. We take evenly spaced points along each chromosome (evenly spaced in terms of genetic distances); and each point serves as center for the next window. The number of points (windows) is chosen so that the windows span the entire chromosome, leaving no gaps in between. To simplify subsequent wavelet transform analysis, we also want the number of windows (or bins) equal to a power of two. Starting from the center of each window, we increase the window until the mean PC1 coordinates for the parental populations are separated by three standard deviations from each mean. The goal is to achieve a complete separation of the parental populations within each window, so there is no ambiguity in assigning chromosomal segments in an admixed genome to either ancestral population. Because human populations are closely related, there is an obvious trade-off between the signal resolution and uncertainty in ancestry estimation; by making the size of the window variable and dependent on the number of informative sites within a given chromosomal region, we always find the smallest possible window that gives us optimal signal resolution without introducing excess errors into the ancestry estimation. Using PA1 coefficients as weights, we find the average value of SNPs within each window. The resulting values are then normalized, so that the ancestral populations correspond to values with means of 1 and -1, respectively. Thus, for each individual we obtain a value for each of the windows, and the windows are evenly spaced along the chromosome. For an admixed individual, the value in each window will either correspond to one of the ancestral populations, or have an intermediate value corresponding to having one chromosomal segment from each ancestral population (we use unphased data, as phasing at the level of an entire chromosome infers haplotypes with significant phasing (switch) errors [14,21], making such data unusable for time since admixture estimation). Thus for each individual and each chromosome we obtain a StepPCO signal, consisting of a sequence of values along the given chromosome. This part of the method is similar to a recently published approach [10], in which local genomic admixture estimates are inferred using PC analysis on a grid of points along the genome (and not genome-wide); unlike our method this approach works with very small windows of 15 SNPs, and requires a Hidden Markov Model (HMM) to infer ancestry state within each window. Our implementation is also different in that we not only estimate the local genomic level of admixture, but also use the identified ancestry block structure to date admixture events.

As mentioned earlier, because most SNPs are not fixed between human populations, it is necessary to use relatively large windows in order to have enough power to reliably assign chromosomal segments to an ancestral population. Large windows mean that the exact location and width of ancestral blocks in empirical data is difficult to determine, because small but informative blocks may be missed, while larger blocks that are actually noise may be inflated and falsely considered a true signal. Therefore rather than to attempt a direct estimation of the number of breakpoints [17] we have developed a method based on spectral analysis of the signal using Haar wavelets [22]. The wavelet transform represents the StepPCO signal (described above), as the sum of simple waves, each characterized by frequency (or period), and position. These wave frequencies are then used as a measure of the width of the ancestral blocks. There are several advantages to the wavelet transform approach. First, wavelet transform of the discrete signal is lossless, and describes the data completely [22]. That is, wavelet transform coeffiicients could be used to recover the original signal exactly. Second, wavelet transform also allows for the reduction of noise, which in this context is defined as high frequency or low amplitude oscillations that are not informative but may falsely be considered as true signals. By removing from the analysis wavelet coefficients corresponding to the high frequency or low amplitude waves within the signal, we are able to greatly reduce the noise and distill the admixture signal contained within the data. Finally, the dominant frequency present in the signal is related to the average width of the admixture blocks, and can therefore be used to infer the time of admixture (see Materials and Methods, Wavelet Transform section for details).

Since the recombination rate is uneven along the chromosome, with 80% of recombination events in humans occurring within hotspots [23], to measure distances along the chromosome we use genetic map distances (measured in cM) rather than physical distances (measured in base pairs). We interpolate genetic distances from genome-wide recombination rates estimated as part of the HapMap project [24].

### Simulations

Initial validation of the method was done using an in-house forward simulation approach. We start with two distinct populations (A and B), simulating chromosomes as an interval from zero to one. We choose the recombination rate to be 2.78 events per chromosome per generation, which corresponds to the recombination rate observed for human chromosome 1 [25,26]. At time *T*_0 _the effective population size of population A equals 1,000 individuals, and it receives either 1%, 5%, 10%, 20%, 30% or 40% migrants from population B. The simulation then runs forward for 2,000 generations; the population at each generation is split randomly into pairs and each pair produces a random number of off spring, drawn from the Poisson distribution, with the average depending on the specified growth rate. The growth rate is chosen so that the population grows from an effective size of 1,000 to 10,000 in 2,000 generations. Since we are only interested in the dynamics of recombination, we only keep track of the recombination points, with their coordinates along the chromosome given as percentages of the total length of the chromosome. This significantly reduces computational time and makes modeling of the recombination dynamics feasible. We ran independent sets of simulations using either the genetic map [25], or the physical position map with variable rates of recombination along the chromosome, using previously-described parameter values for the strength and spacing of hotspots [27]. The recombination map was generated at the beginning of each simulation run. We ran 100 simulations for each of the migration parameters, and from each simulation we sampled 100 chromosomes at exponentially growing time points, and collected statistics on the total amount of admixture, the number of breakpoints, and the width of admixture blocks (as measured by the wavelet transform coefficients) for each chromosome in each sampled generation.

**Figure 2:**
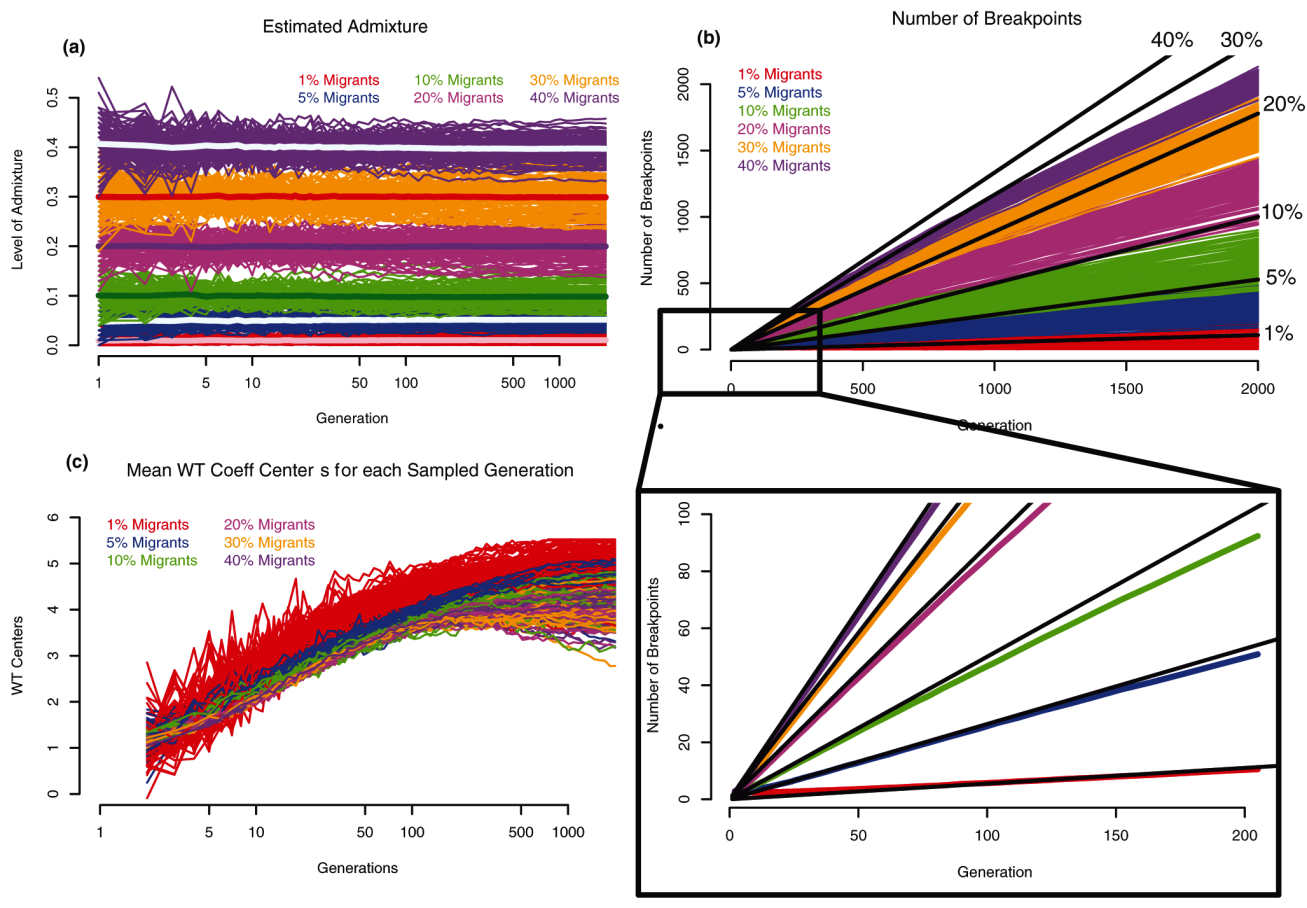
**Data from 100 simulations for migration values of 1%, 5%, 10%, 20%, 30%, and 40%**. Each curve represents a single admixed population. To generate the plots, 100 chromosomes were sampled from each population at exponentially growing time points, and the following statistics for each chromosome in each sampled generation were collected: **(a) **admixture rate; **(b) **number of breakpoints; black lines indicate the expected value, given by: *N*_bkpts _= 2*T*_gen_*R*(*Eα*(1 - *Eα*) - var *α*); and **(c) **the WT centers. Inset: Average number of breakpoints for each simulation parameter. Black lines indicate the expected value.

The overall admixture rate estimates we obtain are highly concordant with the migration rate parameter initially set for each simulation, and this estimation is not influenced by the time since admixture (Figure 2a).

The number of breakpoints vs. time (Figure 2b) is almost linear, with little oscillation within each simulation. There seems to be a stochastic period immediately following the admixture event, when random processes appear to strongly influence the slope. In general, the number of breakpoints grows faster with higher admixture rates (Figure 2b). Up to about 50 generations, the number of observed breakpoints closely matches the expected value:

(1) $$N_{\text{bkpts}}\text{=2}T_{\text{gen}}R\text{(}E\alpha \text{(1 – }E\alpha \text{) – var }\alpha \text{),}$$

where *N *stands for the number of breakpoints, *T*_gen _denotes time since admixture in generations, *R *corresponds to the number of recombination events per generation, *α *denotes the admixture rate for an individual, and *Eα *and var *α *are the mean and the variance of *α*. The deviation of the observed number of breakpoints from that expected after 50 generations (Figure 2b) is due to the fact that infinite populations the pattern of ancestral blocks (their width and distribution along chromosomes) becomes more uniform with time, that is the recombination events are no longer independent.

**Figure 3:**
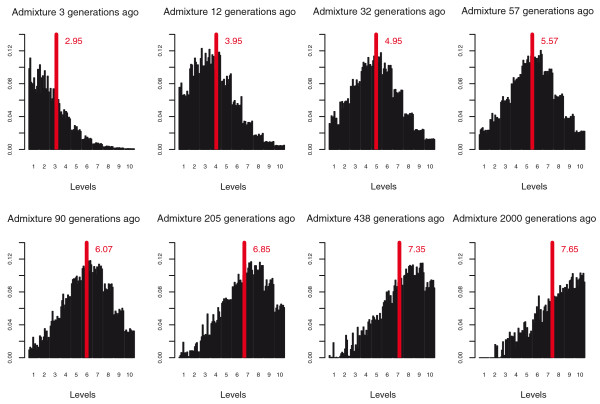
**Distributions of the WT levels, illustrating how the wavelet transform spectrum changes with time since admixture**. For each illustrated time point, WT levels from 10 randomly chosen simulations are plotted (each bar represents one simulation, resulting in 10 bars for each level). The height of the columns indicate the abundance of wavelets of particular frequency present in the signal, starting with the lowest wave frequencies (widest recombination blocks) on the left and progressing towards the highest wave frequencies (narrowest recombination blocks) on the right. The WT centers in this plot are not adjusted for chromosome length, and thus appear to be higher than the values we present for genomewide data.

For the calculation of the wavelet transform (WT) coefficients, simulated chromosomes were randomly paired to form diploids to match the empirical data. Also, from the calculated WT coefficients we exclude all coefficients describing high frequency wavelets (WT levels higher than level seven, as described in the Materials and methods, Wavelet transform section) and normalize for the length of the chromosome by subtracting the log of the chromosome length, which would correspond to the threshold and normalization imposed on the empirical data for chromosome 1 (as the simulated chromosomes were of the same length as chromosome 1). The distributions of the WT levels, indicating how the wavelet transform spectrum changes with time since admixture, are presented in Figure 3. With time the center of the WT spectrum shifts from left to right, from predominantly low to predominantly high frequency wavelets. In Figure 2c the WT centers calculated for different time points are plotted against time since admixture. The centers increase exponentially with time, are fairly independent of admixture rate, and are very consistent across simulations, especially if the admixture rate is over 1%. This measure starts to level off at approximately 400 generations since admixture, which is due to the elimination of levels containing wavelets of highest frequency (done to concur to the filtering applied to the empirical data). For the empirical data, this removal of high-frequency wavelets is done to remove noise, which in turn reflects the relatively low density of informative SNPs present in the data; with more dense SNP data (or full sequence data), we would expect to have more power to detect more ancient admixture events.

### Sensitivity of the method to smaller effective population size and continuous migration

To test the sensitivity of our method with respect to the initial effective population size, we ran additional simulations where the effective size of population A at *T*_0 _equals 500 or 200 individuals, and it receives either 5%, 10% or 20% migrants from population B. The growth rate of the new admixed population was chosen so that in 2,000 generations the population grows to 10,000 or 2,000 individuals, respectively. Results are shown in Figure S1 in Additional file 1. There is no influence of initial population size on the performance of the method for admixture times up to about 20 generations ago. For populations with small Ne and admixture events older than 20 generations ago, our approach will tend to overestimate the date of admixture for more recent events, and not be able to detect more ancient events. Apparently, the diversity in the distribution of ancestry blocks diminishes (stabilizes) faster in smaller populations, thus making new recombination events undetectable. The same phenomenon is responsible for the deviation of the number of recombination breakpoints observed in our simulations from the value predicted by Equation 1. Our results further suggest that it is not so much the small Ne, but rather the growth rate of the population, which is primarily responsible for these deviations. Moreover, the effect is more pronounced when the admixture rate is low.

Additionally, we ran simulations to test how continuous admixture over time affects the method. Again we start with a population A, which at *T*_0 _comprises 1,000 individuals, and it receives either 5% or 20% migrants from population B over the period of either 10 or 30 generations. The growth rate of the new admixed population was chosen so that in 2,000 generations the population grows to 10,000, and 100 simulations were performed for each scenario. Results are shown in Figure S2 in Additional file 1. Because new ancestry blocks are being continuously introduced over the period of either 10 or 30 generations, potentially removing older block structure by replacing narrower ancestry blocks with new wider blocks, we expect ongoing admixture to reduce the wavelet transform coefficients and therefore lead to an underestimation of time since admixture. This is indeed what we observe: irrespective of the admixture rate throughout the duration of admixture (10 or 30 generations), the wavelet transform coefficients are lower than those observed in a population with the same admixture rate, but which has experienced not a continuous but a one-time admixture event. Once the influx of new genetic material into the simulated population A stops, the trajectory of growth of the wavelet transform coefficients is slowly recovered.

### Sensitivity of the method to levels of linkage disequilibrium

As described in the Overview of the Method, to measure distances along the chromosome we use genetic map distances (measured in cM) rather than physical distances (measured in bp). As we measure distances in units of recombination frequency, chromosomal regions with high LD, that is low propensity towards recombination, will span smaller distances and be represented by a smaller number of windows, and conversely genomic regions that harbor recombination hotspots will be inflated and represented by a larger number of windows. We therefore do not expect levels of LD to affect our results. To demonstrate this we have measured LD [28] in fixed windows across chromosomes 6 and 8 in three HAPMAP populations: individuals of European ancestry (CEU), the Yoruban (YRI) individuals and the individuals of African ancestry from the Southwestern USA (ASW). Genotype data were downloaded from the International HapMap project home page [29]. The size of the fixed window was chosen as a fraction of a chromosome length to correspond on average to either 500 kb or to 0.5 cM. In accordance with our expectations we observed that the level of LD varies along the chromosome if the distances are measured in base pairs, but does not vary as much if the distances are measured in cM (Figure S3 in Additional file 1).

### Sample size estimation

For some of the populations considered in this study the sample size was limited to 25 individuals. To ensure that the StepPCO method has adequate power, we therefore calculated how large a sample size is required for accurate and reliable estimates of admixture time. We sampled from 1 to 50 individuals at random from one randomly-chosen simulated population at 12 different time points. Average WT centers, based on different sample sizes, were calculated and used to infer time since admixture by comparing the observed WT centers to those obtained using the entire simulated dataset. The results (Figure S4 in Additional file 1) indicate that a sample size of 10 is sufficient for quite accurate time estimation with narrow confidence intervals up to about 200 generations ago. Point estimates become less precise, and confidence intervals become much wider, at time points exceeding 500 generations ago. This is caused by the threshold imposed on the simulated data to concur with the same limitation that is present in the empirical data, due to the elimination of levels containing wavelets of highest frequency (removal of noise, as described above).

### Comparison to HAPMIX: simulated data

Various methods have been developed to quantify the admixture signal along individual chromosomes, such as ANCESTRYMAP [4], SABER [14], LAMP and LAMP-ANC [15], uSWITCH and uSWITCH-ANC [16], and HAPMIX [17]. To test the performance of the StepPCO approach relative to these other programs, we chose to compare the method only to HAPMIX, as this approach has been shown to perform better relative to the other methods in predicting ancestry transitions, especially for smaller ancestry segments which carry information on more ancient admixture events [17].

To compare to HAPMIX, we constructed an artificially admixed dataset from the phased genotypes of the Yoruban (YRI) individuals and individuals of European ancestry (CEU), downloaded from the International HapMap project home page [30]. Forty haploid admixed genomes were constructed as described previously [17], namely for each simulated chromosome we randomly selected one haploid Yoruban and one haploid CEU genome, and built a recombination map by drawing from an exponential distribution with weight *λ*, such that the ancestry switch occurred with probability 1 - e^-λg ^for each distance of g Morgans. Starting at the beginning of each chromosome and at each of the recombination points from the recombination map, we sampled European ancestry with probability *α *and African ancestry with probability 1 - *α*, where the value of *α *was sampled once from a beta distribution with mean 0.20 and standard deviation 0.10, typical for African Americans [17]. We simulated the following values of *λ*: 6, 10, 20, 40, 60, 100, 200, 400. Once an artificial genome was constructed, parental chromosomes were never reused. Pairs of the resulting artificially admixed haploid genomes were merged to create 20 diploid admixed individuals.

We then compared the performance of our spectral decomposition method to HAPMIX on these artificially admixed genomes. We ran a StepPCO analysis, followed by the WT decomposition of the resulting StepPCO admixture signal. To investigate how the dominant wavelet frequency is related to λ for this artificial dataset, we generated a separate dataset of hybrids. Maps of recombination events for each of these additional hybrid genomes for the values of λ: 6, 10, 20, 40, 60, 100, 200, 400 were constructed as described in the previous paragraph. 20 hybrids genomes were constructed for each value of λ, and spectral decomposition analysis was carried out on the resulting admixture signal. Using the known number of breakpoints in these simulated hybrids, we have found that the dominant wavelet frequency is linearly related to the logarithm of the average (per Morgan) number of ancestry switches (breakpoints); linear regression was used to find the coefficients, and estimate the average number of ancestry switches per unit of genetic distance in the main simulated dataset.

**Figure 4:**
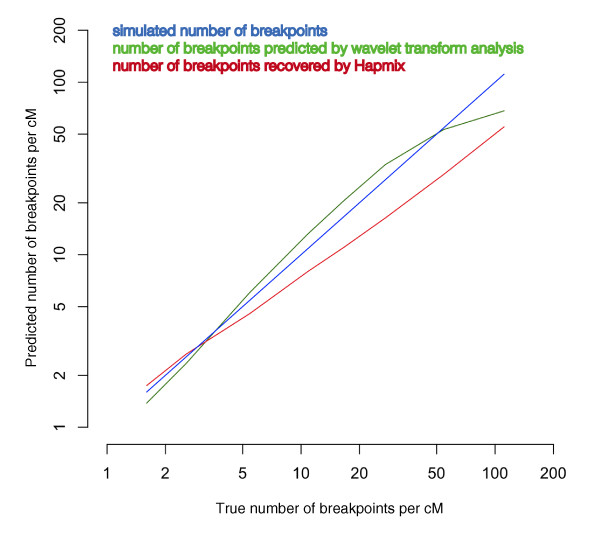
**Performance of Hapmix and wavelet transform analysis in recovering the average number of recombination breakpoints per Morgan of genetic distance from simulated data**. The two methods were applied to 20 artificially admixed individuals, created using a genomewide average of 20% European and 80% African ancestry. For simulated data the average number of ancestry switches (or breakpoints) was drawn from an exponential distribution with weight λ, such that the ancestry switch occurred with the probability 1 - *e*^-λg ^for each distance of g Morgans. The following values of λ were simulated: 6, 10, 20, 40, 60, 100, 200, 400. Since in the simulated genomes the true number of breakpoints is known, we show the accuracy of both methods in recovering this information.

We also ran HAPMIX on the same artificially-admixed samples, using 40 haploid YRI and 40 haploid CEU genomes as the reference parental populations and using the input parameters recommended previously [17]. We calculated the number of ancestry switches detected by HAPMIX, as the output of HAPMIX produces probability associated with each SNP genotype in the admixed genome. We then compared the true number of ancestry switches per Morgan of genetic distance (known for simulated data) to the estimates produced by either HAPMIX or WT decomposition of the admixture signal. The results (Figure 4) show that HAPMIX consistently underestimates the number of breakpoints, while the estimates obtained by the WT analysis are more accurate, especially for higher values of λ, typical of more ancient admixture events.

However, accurate estimation of breakpoints does not imply accurate estimation of the admixture time. As demonstrated previously (Figure 2b), the number of breakpoints can deviate significantly from the value predicted by Equation 1, especially with higher admixture rates, lower ancestral Ne, and/or older admixture times. Furthermore, inference of breakpoints requires transformation of the 'raw' genomic signal into a discrete signal corresponding to the presence or absence of an ancestry switch, hence direct inference of number of breakpoints is inevitably error-prone. These errors, however small, will accumulate over the many measurements taken. WT analysis avoids such errors because rather than inferring the recombination events directly, the WT method compares the spectral properties of the given signal in the observed data with the properties of the model signal produced by simulations.

### Empirical data

Quality-filtered genotypes for approximately one million SNPs for 25 Polynesians (PLY), 25 Fijians (FIJ), 23 Borneans (BOR) and 25 individuals from the highlands of Papua New Guinea (MEL), typed with Affymetrix 6.0 arrays, were obtained from a previous study [19] and are available from the authors upon request. Quality-filtered genotypes obtained with the Illumina Human1 M and Affymetrix 6.0 arrays for 20 Yorubans from Ibadan, Nigeria (YRI), 20 individuals of northern and western European ancestry living in Utah (CEU) and 20 individuals of African ancestry from the Southwestern USA (ASW), were downloaded from the International HapMap project home page [29]. SNPs were merged using the PLINK tool [31], to include only markers which were genotyped and passed the quality filters in both datasets. The final dataset comprised 653,498 SNPs. We also analyzed and dated admixture in Mandenka, Mozabite, Bedouin, Palestinian and Druze groups from the CEPH-HGDP [18]. These groups were previously analyzed via HAPMIX and reported to have European-related ancestry ranging from 2% to 97%, when analyzed using Africans and Europeans from the HapMap as the input reference populations [17]. These samples were genotyped for 650,000 SNPs on the Illumina platform [18]. The data were downloaded from the HGDP CEPH Genotype Database [32].

For the empirical admixture analyses, the parental groups are: the French and Yoruba for the admixed Mandenka, Mozabite, Bedouin, Palestinian and Druze groups; the YRI and CEU groups for the admixed ASW group; the BOR and MEL groups for the admixed PLY group; and the MEL and PLY groups for the admixed FIJ group. The StepPCO approach was first used to elucidate the local structure of the admixture signal for each admixed individual along each chromosome. We then estimated admixture proportions in each admixed group, and compared the StepPCO results for each chromosome to admixture proportions estimated using the maximum-likelihood based algorithm implemented in *frappe *[13]. We then applied wavelet transform analysis to the StepPCO signal and used the wavelet transform coefficients to infer time since admixture. After the wavelet transform coefficients were calculated we applied three filtering procedures to the signal. First, as explained previously, we replaced all coefficients smaller than an ascertained threshold value by zero, to remove low amplitude oscillations that are characteristic of noise (that is, wavelets of low height). This threshold value was chosen so that small oscillations present only within the distribution of the parental individuals are ignored. Second, we removed WT levels that correspond to the wavelets of the highest frequencies, which are also characteristic of noise (that is wavelets that are too narrow). Then we averaged the coefficients across each level and found a threshold amplitude, which is present in every individual whether admixed or not, and consider everything below it as noise (in effect this means that we subtract the parental signal, that is when we analyze the admixture signal in FIJ for example, with PLY and MEL being the ancestral populations, the fact that PLY themselves harbor Melanesian admixture has no effect on the inference of the admixture date for FIJ). Finally, we find the dominant frequency present in the signal (WT center) and use it to infer the time of admixture by comparing this observed dominant frequency to that obtained in simulated data generated using the admixture rate observed in the empirical data.

#### African-Americans, Polynesians and Fijians

**Figure 5:**
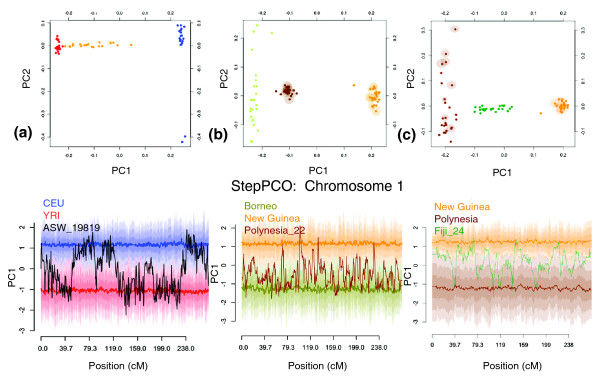
**PCA and StepPCO results for chromosome 1**. Solid lines centered around 1 and -1 indicate the mean PC1 coordinate for each parental population; progressively lighter shading surrounding the mean of each parental group indicate +/-1, +/-2 or +/-3 standard deviations from the mean. **(a) **Upper panel: PC1 vs PC2 for populations of CEU, YRI and ASW. Lower panel: Unphased chromosome 1 of an individual of African American ancestry; European (blue) and Yoruba (red) populations are used as parental groups. **(b) **Upper panel: PC1 vs PC2 for populations of MEL, BOR and PLY. Lower panel: Unphased chromosome 1 of an individual from Polynesia; Borneo (green) and New Guinean (orange) populations are used as parental groups. **(c) **Upper panel: PC1 vs PC2 for populations of MEL, PLY and FIJ. Lower panel: Unphased chromosome 1 of an individual from Fiji; Polynesia (brown) and New Guinean (orange) populations are used as parental groups.

StepPCO plots for one ASW, one PLY, and one FIJ are presented in Figure 5. The pattern of chromosomal segments alternating between two ancestral states is characteristic of all admixed individuals and is observed on all chromosomes. For some chromosomal segments intermediate PCA1 values are observed, indicating that the admixed individual contains chromosomal segments from both parental populations (see Figure S5 in Additional file 1 for StepPCO results for the other chromosomes from these three individuals). As described in the Overview of the Method, the number of SNPs per sliding window of the StepPCO analysis is allowed to vary, in order to achieve reliable assignment of chromosomal segments in admixed individuals to the correct parental group. The average number of SNPs per StepPCO sliding window for chromosome 1, which contained a total of 42,499 SNPs after filtering, was: 280 for African Americans, 519 for Polynesians, and 1015 for Fiji. This variation reflects the different levels of differentiation between the ancestral populations of these three admixed groups. The largest average size of the sliding window is observed for Fiji, where PLY and MEL are used as parental groups. As the PLY themselves have Melanesian ancestry, PLY and MEL are much less differentiated than CEU and YRI, the parental populations of the African Americans. Hence more SNPs are needed to reliably assign chromosomal segments in the FIJ group to either of the ancestral populations, than are needed for the ASW.

**Figure 6:**
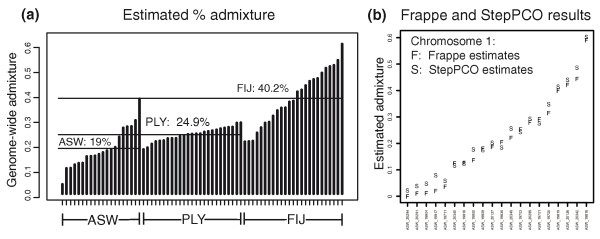
**Admixture estimates**. **(a) **Genome-wide admixture estimates based on StepPCO for African-Americans, Polynesians and Fijians. **(b) **Comparison of admixture estimates obtained via StepPCO vs. Frappe, for chromosome 1 for 20 African-Americans.

Average admixture proportions estimated by the StepPCO method for the African-Americans, Polynesians and Fijians are 19% European ancestry, 24.9% Melanesian ancestry, and 40.2% Melanesian ancestry respectively (Figure 6a). Individual admixture estimates vary substantially among the African-Americans, with some individuals exhibiting very low European ancestry (less than 5%), and some substantially higher (more than 40%). These results were substantiated by the *frappe *[13] analysis, which agree quite closely with the per-chromosome ancestry estimates from the StepPCO analysis (Figure 6b). A similar pattern is observed in Fiji, with Melanesian ancestry ranging from 22% to 63%. Despite the fact that the Polynesian sample is very diverse, coming from seven different islands [19], the level of Melanesian ancestry is much more uniform across individuals (varying from 18 to 28%).

**Figure 7:**
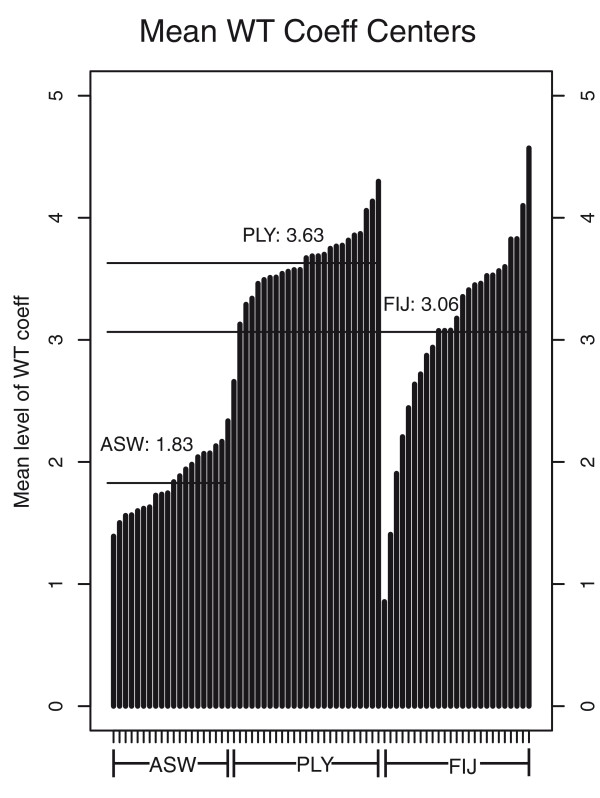
**Average centers of the WT coefficients, calculated for each individual**.

**Figure 8:**
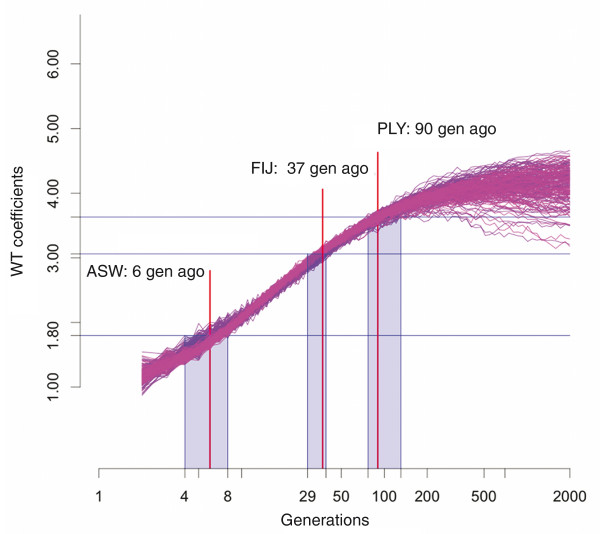
**Admixture time estimates for the African-Americans, Polynesians and Fijians**. Simulated data from 100 simulations with a 20% and 40% migration rate are presented. Each curve represents a single admixed population. Average WT centers calculated for 100 chromosomes drawn at random from each population at exponentially growing time points are plotted as a function of time. Measurements obtained for the ASW, PLY and FIJ populations are shown by blue horizontal lines. Red vertical lines indicate the time estimate, and shaded boxes define the confidence intervals. Time estimate for ASW and PLY are based on simulations with a 20% admixture rate, while the time estimate for FIJ is based on simulations with a 40% admixture rate.

The spectral analysis of the StepPCO signal revealed that the average dominant frequency for the African-Americans is located at level 1.8, which would correspond to an abundance of low frequency wavelets (that is, wider ancestry blocks), while for the Fijians and the Polynesians the average dominant frequency is at level 3.06 and 3.63 respectively, which is indicative of much narrower ancestry blocks (Figure 7). Based on simulations, the WT center of 1.8 corresponds to an admixture time of 6 generations ago (95% CI: 4-8 generations) for the African Americans. Assuming a generation time of 30 years [33], our results indicate that the admixture in the African Americans started about 180 years ago. Similarly, the simulations indicate that the WT center of 3.63 for the Polynesians corresponds to an admixture time of 90 generations (95% CI: 77-131 generations), or about 2,700 years ago (Figure 8). The time estimation for Fiji is based on simulated data with a 40% admixture rate (to match the higher admixture rate of Fiji), and here the WT center of 3.06 corresponds to an admixture time of 37 generations (95% CI: 29-39) or about 1,100 years ago.

#### HGDP populations

To test how the performance of our approach compares to HAPMIX, we applied our method to the Mandenka, Mozabite, Bedouin, Druze and Palestinian populations from the CEPH-HGDP [18], which were previously analyzed using HAPMIX [17]. The HAPMIX estimates for the European-related ancestry in these populations ranged from 2% to 97%, when analyzed using Africans and Europeans from the HapMap as the input reference populations (Table 1). The time since admixture in these populations was inferred by calculating the number of genomewide ancestry transitions (or the number of breakpoints), and the results are reported in Table 1.

**Table 1:** Comparison of results from HAPMIX and StepPCO

Population	Estimated percent European ancestry from HAPMIX	Estimated percent European ancestry from StepPCO	Estimated time since admixture (in gens ago) from HAPMIX	Estimated time since admixture (in gens ago) from StepPCO
Mandenka	2%	2%	120	121
Mozabite	77%	84%	100	131
Bedouin	91%	91%	90	83
Palestinian	93%	92%	75	72
Druze	97%	96%	60	90

We have removed one outlier individual from the Mandenka population. Among the Bedouins, 24 out of 45 individuals have a higher proportion of European-related ancestry (Figure S7 in Additional file 1). If these individuals are removed from the analysis, the estimate for the admixture time in the Bedouins changes to 97 generations ago. Estimated percent European ancestry was calculated by using the two parental populations and an admixed group to find the PC1 axis.

Although we estimated similar admixture proportions for these populations (Table 1), subsequent spectral analysis of the admixture signal in the Mandenka, Mozabite, Bedouin, Palestinian and Druze revealed older admixture dates for the Mozabites and the Druze populations (Table 1 and Figure S6 in Additional file 1). The Bedouin population appears to be structured, with 24 out of 45 individuals having a much higher proportion of European-related ancestry (Figure S7 in Additional file 1). If these individuals are removed from the analysis, the estimate for the admixture time in the Bedouins changes to 97 generations ago (CI: 83-131).

All programming and data analysis was performed using R (ver. 2.10.1) [34]. All scripts are freely available [35].

## Conclusions

Using genetic data to infer the time of migrations has always been difficult, and the time estimates obtained often come within wide confidence intervals, making these dates unreliable and inferences problematic. Here, we have introduced an approach that takes advantage of dense genome-wide SNP data to improve precision and reduce bias in making inferences about the timing of human migrations. By using an admixed population one can capitalize on the property of the genome to recombine each generation, producing chromosomes that are a mixture of the parental genetic material. The structure of an admixed genome contains temporal information about an admixture event, as a greater number and narrower width of ancestry blocks indicates more recombination events, and hence greater time depth.

Simulations indicate that the WT coefficients can be used to obtain accurate estimates of the time of admixture from suitable genome-wide SNP data. We therefore applied the method to three datasets, consisting of about 650,000 SNPs, to estimate the amount and time of admixture for three human populations: African-Americans, Polynesians, and Fijians. In addition, we analyzed and dated admixture in five HGDP populations of African and Middle-Eastern origin. At first glance, it may appear that the simulated and empirical data differ in that the simulations used fully-differentiated populations, which is not the case for the empirical data. However, as explained in more detail in the Results and Discussion (Basic Setup section), the number of SNPs is adjusted in the StepPCO sliding windows until the ancestral populations can be statistically-differentiated, just as with the simulated data.

For African-Americans, we estimate an average of 19% European ancestry, with a wide range of less than 5% to more than 40% European ancestry across individuals. Both the average and the observation of a wide range of individual admixture estimates are in keeping with previous studies [10,17,36,37]. The estimated time of admixture is about 180 years ago (95% CI: 120-240 years ago), which is probably an underestimate since admixture in the African-American population is ongoing (implying that new ancestry blocks are being continuously introduced by new recombination events, which potentially removes older block structure by replacing narrower ancestry blocks with new, wider blocks).

We tested the performance of the method on Fijians and Polynesians, as both populations are of admixed Asian and Melanesian ancestry [6]. Previous demographic analyses of the genome-wide SNP data used in this study strongly support both an admixed Asian/Melanesian ancestry for Fijians and Polynesians as well as subsequent additional gene flow from Melanesia into Fiji, but not Polynesia [19]. Based on this previously established scenario, we estimated an average of about 25% (from 18 to 28%) Melanesian ancestry in Polynesians, in good agreement with previous estimates based on the same [19] or other [6,38-40] data. The estimated time of admixture is about 90 generations ago, or 2,700 years (95% CI: 2,300-3,900 years), in good agreement with a previous estimate of about 3,000 years ago based on an ABC simulation approach for the same data [19]. For Fiji, the estimated amount of Melanesian ancestry was about 40%, and the time for this admixture is estimated to have occurred about 37 generations ago, or 1,100 years (95% CI: 870-1170 years). An ABC-simulation based approach for the same data gave an estimated date of 62 generations for this admixture in Fijians, about twice as long ago as our estimate. We speculate that, as in the case of the African-Americans, the estimate based on WT coefficients may be biased toward more recent dates if the gene flow to Fiji occurred over a period of time, as more recent gene flow replaces older, narrower ancestry blocks with newer, wider ancestry blocks. Individual Melanesian ancestry estimates are much wider for Fiji (from 22 to 63%) than for Polynesia (from 18 to 28%), which may indeed indicate a longer period of gene flow into Fiji.

Our results for the Mozabite, Mandenka, Bedouin, Druze and Palestinian populations are similar to those for HAPMIX for inferring local ancestry, and in addition our method seems to perform better with respect to more ancient admixture events (as also shown with simulated data: Figure 4). In particular, we dated the admixture event in the Mozabites and the Druze to 131 and 90 generations ago respectively, 30 generations more than the corresponding estimates obtained with HAPMIX [17]. HAPMIX estimation of the time since admixture is based on the number of calculated ancestry transitions (that is, the number of breakpoints); both our simulations and previous simulations [17] indicate that infinite size populations the number of breakpoints does not increase with time according to expectations (see Equation 1 above), but rather stabilizes, leading to underestimates in admixture dates (Figure 2b). Furthermore, because human populations are closely related and not very well differentiated, direct estimation of the number of breakpoints and block width as a measure of time since admixture for human genetic data is problematic for two reasons. Firstly, to have enough power to reliably assign chromosomal segments to an ancestral population, it is necessary to use relatively large genomic windows, which correspondingly reduces detection of closely-spaced breakpoints. And secondly, for every location in the genome that potentially carries a breakpoint, a formal decision has to be made as to whether to consider it a true breakpoint or not. This transformation of the 'raw' signal into a discrete signal potentially leads to either some not well-defined breakpoints being overlooked, or conversely random effects becoming inflated and falsely considered as a true signal. These errors, however small, will accumulate over the many measurements taken. Conversely, the spectral analysis approach implemented here does not require any data transformation and is applied to the 'raw' signal directly. This has the advantage of preserving the statistical nature of the signal until the final averaging step, and thus does not involve detection of exact location (and presence) of breakpoints, where inevitably large errors in estimation could occur. Although we followed Price *et al*. 2009 in using African and European parental groups for the admixed Mozabite, Mandenka, Bedouin, Druze and Palestinian groups from the CEPH-HGDP, in fact previous studies have shown that the Druze, Bedouin and Palestinian populations are admixed primarily along a European-Central Asian axis, with little African admixture, and the Mandenka exhibit very little European admixture [18,41]. Here, we report dates for the presumptive European gene flow, to compare our results to the previous study [17], but it is important to keep in mind that our method (like all admixture methods) requires the use of pre-defined parental groups. Incorrect identification of the ancestral groups contributing to an admixed group will obviously lead to erroneous conclusions, hence careful attention must be paid when identifying parental groups. This is especially true for groups that are suggested to have experienced admixture a long time ago, and hence had more time to experience genetic drift (which is always expected to act in a direction orthogonal to the axis of admixture). In such cases, it is difficult to distinguish between an admixed population that has been subject to genetic drift, and a population that has experienced admixture along a different axis of variation.

Theoretically, there are no limitations as to how far back in time one can get good estimates of admixture time with WT coefficients. The performance of the method is influenced by two factors: the density of SNPs analyzed and the degree of differentiation between the two parental populations. Increasing SNP density would allow the estimated time horizon for detecting admixture to be moved further back. We therefore expect that full sequence data will increase the sensitivity and resolution of our method. The analysis presented here was based on about 650,000 markers; the current estimates for the number of SNPs in the human genome is around 15 million SNPs [42]. Full sequence data will thus provide a twentyfold increase in SNP density, and thereby allow for a twentyfold reduction in the size of the sliding window. Thus, assuming that the newly added SNPs are no less informative for population differentiation than the SNPs on the Affymetrix arrays, we expect that analysis of full sequence data should offer at least a twentyfold improvement in the potential time depth for admixture estimates for human populations. However, given that there is relatively little genetic differentiation between human populations, to distinguish among parental populations requires relatively large segments of the genome, and this also poses a restriction on the time depth of the method. The more closely-related the parental populations, the larger the window size needed to span a sufficient number of informative SNPs. Obviously, this limitation will persist regardless of the type of molecular data considered. However, because of the strong ascertainment bias associated with the SNPs genotyped on the arrays, we expect that SNP-data generated using the array technology necessarily underestimates the variation that exists between human populations, and recent studies suggest that this underestimation could be considerable [43]. Moreover, the method introduced here can be used with any species for which suitable genome-wide data exist, and the larger the genetic difference among the parental populations, the less genome-wide data needed for accurate admixture estimates.

An additional advantage of the StepPCO method is that it provides an estimate of the admixture proportions for each individual within an admixed population. Individual-level estimates of admixture obtained here via StepPCO for African-Americans, Polynesians, and Fijians were quite similar to those obtained via the maximum-likelihood based approach *frappe *[13], indicating that StepPCO gives reliable results. Furthermore, the StepPCO method also provides information about the distribution of admixture along each chromosome. As such, this approach is also promising for disease gene mapping (in recently admixed populations), and for studying local selection. According to neutral expectations the admixture level should be constant along the genome, but a locus favored by positive selection in the admixed population should appear to have greater admixture proportions than would be expected from the genome-wide average. We are currently investigating the utility of this approach for identifying candidate genes subject to local selection.

In conclusion, we have shown that wavelet transformation is a useful and novel means of dating admixture events from genome-wide data. Other potential extensions of the methodology introduced here include admixture scenarios involving more than two parental populations, and implementing spectral analysis of the raw genomic signal directly, rather than from the StepPCO signal. There is potentially much more to be learned from surfing the wavelets of the genome.

## Materials and methods

### Basic setup

We consider a collection of *N *SNPs along a chromosome, which are ordered by their physical position, and indexed by numbers from 1 to *N*. For such a collection of SNPs we consider a vector $\text{p}\;\text{:}={\left(p_{i}\right)}_{i=1}^{N}$ of each SNP's physical position along the chromosome scaled with respect to the genetic distances, which were interpolated from genome-wide recombination rates, estimated as part of the HapMap project [24]. Suppose **e **is an individual chromosome. We also denote $\text{e}\;\text{:}={\left(e_{i}\right)}_{i=1}^{N}$ as a collection of calls at given SNP positions for the chromosome **e**. Each component in the vector **e **for each individual chromosome takes value 0, 1, -1 or **NA**, where -1 and 1 are assigned to the two possible homozygotes of a given SNP and 0 corresponds to a heterozygote. The space of all possible calls (excluding **NAs**) sits as a discrete set in an *N*-dimensional real vector-space *E *with canonical basis.

We then consider a sliding window along each chromosome. Such window **w **refers to a contiguous subrange of SNPs, that is:

$$\text{w}=\left\{w_{\text{first}},w_{\text{first}}+1,...,w_{\text{last}}-1,w_{\text{last}}\right\}.$$

The width of a window is defined by the genetic distance between the last and the first SNP in the window:

$$\text{Width}\left(\text{w}\right):\;=\;p_{w_{\text{last}}}-\;p_{w_{\text{first}}}.$$

Suppose we are given an oriented line *A *in the vector-space *E *(later *A *will be taken to be a principal axis of a certain collection of individual chromosomes). Line *A *is spanned by a single non-zero vector with coordinates ${\left(a_{i}\right)}_{i=1}^{N}$ defined up to multiplication by a positive number.

For a given window **w **and an individual chromosome **e**, define a measurement associated with this window with respect to the axis *A *by:

$$M_{\text{w}}\left(\text{e}\right)\;\;:\;=\;\frac{\sum_{i\in \text{w}}a_{i}e_{i}}{\sum_{i\in \text{w}}\left|a_{i}\right|}..$$

Obviously, the resulting value does not depend on the choice of a vector spanning *A*.

Given a point *x *along the chromosome, we say that a window **w **is centered at a point *x *and has a width *l *if it consists of exactly those SNPs that lie within the distance *l*/2 from *x*, that is:

$${\text{w}}_{l}(x):=\left\{w||x-p_{w}|\leq \frac{l}{2}\right\}.$$

Consider sample collections {**p***^k^*} and {**q***^k^*} from two populations *P *and *Q*, respectively. Let $\text{a}={\left(a_{i}\right)}_{i=1}^{N}$ define the principal axis in SNP-coordinates for a collection of samples {**p***^k^*,**q***^k^*}. Given a point *x*_0 _along the chromosome and a window **w **centered at this point, consider measurements:

$$\alpha_{k}:=M_{\text{w}}(p_{k})\;\text{and}\;\beta_{k}:=M_{\text{w}}(q_{k}).$$

Given a positive real number *λ *> 0, we say that populations *P *and *Q *are *λ*-separated in a window **w **if:

(2) |Eα−Eβ| ≥λ|σ(α)+σ(β)|,

where E stands for the mean and *σ *for the standard deviation estimators of the PC1 coordinate of populations *P *and *Q*.

Finally, a window **w **is called *λ*-optimal if it has the smallest size, such that populations *P *and *Q *are *λ*-separated in **w **(that is the smallest possible window that satisfies Condition 2). The size of this window at each position is chosen so that the ancestral populations are sufficiently well separated by the statistical properties of the collection of SNPs in the window. We set *λ *= 3, that is we consider a window as optimal, when the mean PC1 coordinates for the parental populations are separated by three standard deviations from each mean. Note that in general the optimal size will depend on the position of the window along the chromosome. In effect, by taking smaller windows in chromosomal regions that contain more informative SNPs, we are able to increase signal resolution without introducing excess errors into the ancestry estimation.

In summary, for the sample collections {**p***^j ^*} ⊂ *P *and { **q***^j ^*} ⊂ *Q *we construct the following data: a) the principal axis A spanned by a non-zero vector with coordinates ${\left(a_{i}\right)}_{i=1}^{N}$ for a collection of samples {**p***^j^*, **q***^j^*}; b) a collection of *K *equispaced points ${\left\{x_{k}\right\}}_{k=1}^{K}$ along the chromosome; and c) for each point *x_k_*, *k *= 1,..., *K *we construct a *λ*-optimal window **w***_k _*centered at *x_k_*. These data we refer to as a StepPCO frame. (Special care needs to be taken so that the windows cover the entire chromosome, leaving no gaps in between. This can always be achieved by choosing the number of bins *K *sufficiently large.)

### StepPCO

Using coefficients of PA1 as weights, we find the average value of SNPs within a window. The resulting values are then normalized, so that the ancestral populations correspond to values with means of 1 and -1, respectively.

Consider collections {**p***^j ^*} and { **q***^j ^*} of samples from two ancestral populations *P *and *Q *and a set of admixed individuals *R *= {**r***^j^*}. Construct a StepPCO frame (in our applications *λ *= 3 and number of bins is *K *= 1,024).

For an individual chromosome **r **from population *R*, define the StepPCO signal as a vector of measurements:

$$S(\text{r}):={\left(M_{{\text{w}}_{k}}(\text{r})\right)}_{k=1}^{K}.$$

Each component of the vector S(r) characterizes a stretch of chromosome **r **corresponding to each bin as belonging to one of the ancestral populations, and the confidence of such an assignment. The level of confidence will of course depend on *λ*.

Suppose the principal axis is directed from *P *to *Q*. Then the closer the value of the signal to 1, the more likely that the corresponding stretch of the chromosome of the sample **r **belongs to ancestral population *Q*.

Analogously, values close to -1 correspond to the stretches of DNA most likely tracing their ancestry to population *P *.

### Wavelet transform

Consider a 2*^L^*-dimensional vector-space $V={\mathbb{R}}^{2^{L}}$ with the scalar product:

$$\langle \left(u_{i}\right),\left(v_{i}\right)\rangle :=\sum_{i=1}^{2^{L}}u_{i}v_{i}.$$

Wavelets (ω*_l, p_*) are the orthogonal system of 2*^L ^*- 1 vectors in *V *. They are indexed by the level *l *= 1,..., *L *and the position *p *= 1,..., 2^*l*-1^. The level corresponds to a particular frequency of the wave, and is up to an additive constant the negative logarithm base 2 of the period (see formula 3), while the position denotes the position of the wavelet of the given frequency within the signal. Each wavelet at level *l *has a support of the size *2*^*L-l+*1 ^and is a rectangular wave of amplitude 1 and zero average.

The coefficients of a wavelet (ω*_l, p_*) are given by:

$${\left(\omega_{l,p}\right)}_{k}:=\{\begin{array}{ll} 1 & \text{if (}p-1\text{)}2^{L-l+1}+1\leq k\leq (p-1)2^{L-l+1}+2^{L-l},\\ -1 & \text{if (}p-1\text{)}2^{L-l+1}+2^{L-l}+1\leq k\leq p2^{L-l+1},\\ 0 & \text{otherwise}\text{.} \end{array}$$

Together with the vector with all coefficients equal to 1, wavelets form an orthogonal basis of *V*. Suppose $\gamma ={\left(\gamma_{i}\right)}_{i=1}^{2^{L}}$ is a signal with a discrete time, that is a 2*^L^*-dimensional real vector from *V*. Its wavelet coefficients are simply coefficients of *γ *with respect to the wavelet basis. They could be efficiently evaluated by passing *γ *through a series of filters (linear operators) obtaining at each step: i) wavelet coefficients for a given level, and ii) a downsampled signal to which the next round of evaluation is to be applied:

$$\begin{array}{l} \text{For}\;k=1,...,2^{L-1}\{\begin{array}{ll} {\gamma^{\prime }}_{k}:=\frac{1}{2}(\gamma_{2k}+\gamma_{2k-1}) & \text{low}\;\text{pass}\;\text{filter,}\\ {\text{wt}}_{L,k}(\gamma ):=\frac{1}{2}(\gamma_{2k}-\gamma_{2k-1}) & \text{high}\;\text{pass}\;\text{filter,} \end{array}\\ \text{For}\;k=1,...,2^{L-2}\{\begin{array}{ll} {\gamma^{\prime \prime }}_{k}:=\frac{1}{2}({\gamma^{\prime }}_{2k}+{\gamma^{\prime }}_{2k-1}) & \text{low}\;\text{pass}\;\text{filter,}\\ wt_{L-1,k}(\gamma ):=\frac{1}{2}({\gamma^{\prime }}_{2k}-{\gamma^{\prime }}_{2k-1}) & \text{high}\;\text{pass}\;\text{filter}\text{.} \end{array}\\ \;\;\;\;\vdots \;\;\;\;\;\vdots \end{array}$$

As a result, one obtains 2^*L*-1 ^wavelet coefficients and one additional value: of the last downsampled signal (*γ*_average_). This *γ*_average _corresponds to the average of *γ*. Note that the level *l *of a wavelet is defined as the logarithm base 1/2 of the halfperiod *p *of the wave, where period is measured as a fraction of the length of the entire signal:

(3) $$p=\frac{1}{2}\frac{2^{L+1-l}}{2^{L}}=2^{-l}\;\text{or}\;l={\text{log}}_{\frac{1}{2}}p.$$

Thus, to compare wavelet coefficients of signals corresponding to some physical phenomena, levels should be shifted by the logarithm base 1/2 of the physical length of the signal.

Wavelet transform of the discrete signal is lossless [22], and the original signal could be recovered from its wavelet coefficients and its average, as:

$$\gamma \;\text{=}\;\gamma_{\text{average}}\left(\begin{array}{c} 1\\ \vdots \\ 1 \end{array}\right)+\sum_{l,k}{\text{wt}}_{l,k}\left(\gamma \right)\omega_{l,k}.$$

We will refer to this as inverse wavelet transform and write:

$$\gamma \text{=iwt(}wt_{l,k},\gamma_{\text{average}}\text{)}.$$

For the noise reduction, one decides which wavelet amplitudes at each frequency and location are to be considered unimportant, and filters the wavelet coefficients removing (setting to zero) corresponding coefficients. Inverse wavelet transform then produces a signal with the 'noise' removed. An example of this procedure is shown in Figure S8 in Additional file 1 where random noise is added to the signal and then removed using procedure described above.

That is, given a collection **t **= (*t_l, k_*) of threshold levels, one for each of the wavelets in the wavelet decomposition, define a noise filter T on the set of wavelet coefficients by:

$$\begin{array}{c} \tilde{\text{wt}}=T(\text{wt}),\;\text{where}\\ {\tilde{\text{wt}}}_{l,k}=\{\begin{array}{ll} 0 & {\text{if |wt}}_{l,k}\text{|}\;\leq t_{l,k},\\ {\text{wt}}_{l,k} & \text{otherwise}\text{.} \end{array} \end{array}$$

Given a signal containing noise, one finds its wavelet coefficients, applies a noise filter T to the set, and uses an inverse wavelet transform to recover the signal without the noise. The cleaned signal then is:

$$\gamma_{\text{cleaned}}=\text{iwt(}T(\text{wt(}\gamma \text{)})\text{)}.$$

To assess the spectral characteristics of the signal we use the following procedure: Suppose *γ *is a discrete signal as above, and wt = (wt*_l, k_*) is the collection of its wavelet coefficients. First we calculate the so called wavelet summary by averaging absolute values of wavelet coefficients at each level. That is:

$$s_{l}:=\frac{\sum_{k=1}^{2^{l-1}}\left|{\text{wt}}_{l,k}\right|}{2^{l-1}},\;l=1,\ldots L.$$

Each (non-negative) number *s_l _*shows the abundance of the corresponding wavelet frequency in the signal. Finally, the dominant frequency present in the signal, so called center, is evaluated as follows:

$$C(\gamma )=\frac{\sum_{l=1}^{L}\left(l\cdot s_{l}\right)}{\sum_{l=1}^{L}s_{l}}.$$

Thus, C(*γ*) is a 'central' frequency of the signal *γ*, and is used as an indirect measure of the average width of the admixture blocks.

## Abbreviations

ASW: African Ancestry in SW USA; BOR: Borneo; CEU: U.S. Utah residents with ancestry from northern and western Europe; FIJ: Fiji; HGDP: Human Genome Diversity Panel; HMM: Hidden Markov Model; MEL: the highlands of Papua New Guinea; PA1: first principal axis; PCA: principal component analysis; PLY: Polynesia; WT: wavelet transform; YRI: The Yoruba in Ibadan, Nigeria.

## Authors' contributions

IP, RM and MS conceived and designed the experiments. IP and RM performed the experiments. IP analyzed the data. RM and IP contributed analytical tools. AW and MK contributed data. IP, RM and MS wrote the paper.

## Supplementary Material

Additional file 1 **Supplementary figures**. Additional file includes eight supplemental figures. Click here for file
