# Supplementary material for: Dating the age of admixture via wavelet transform analysis of genome-wide data
Source: Genome Biol. 2011 Feb 25;12(2):R19. doi: 10.1186/gb-2011-12-2-r19 (PMC3188801; doi:10.1186/gb-2011-12-2-r19)
Supplement: Additional file 1 — Supplementary figures. Additional file includes eight supplemental figures. [file gb-2011-12-2-r19-S1.PDF]

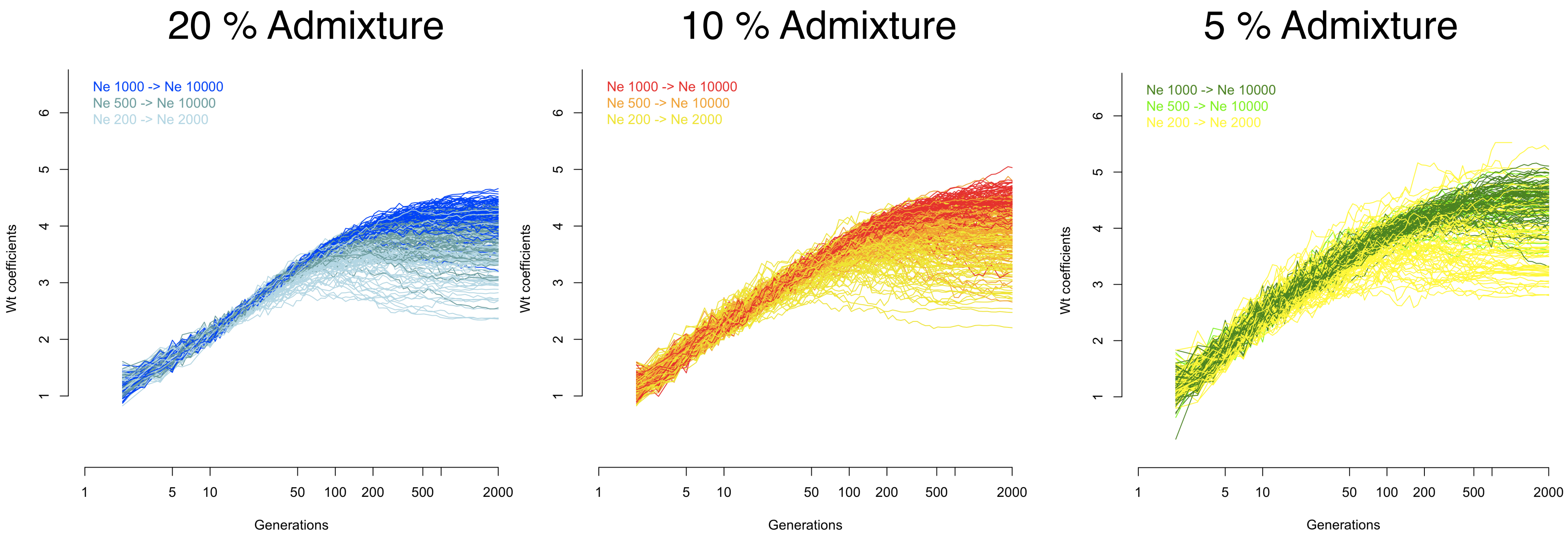

Figure S1. – Sensitivity of the StepPCO method with respect to the initial effective size of the population. Shown data are from 100 simulations for the initial Ne of 1000, 500 and 200 individuals, and population growth over 2000 generations to 10,000, 10,000 and 2,000 individuals respectively. The three panels show simulations performed with the migration values of 5%, 10% and 20%. Each curve represents a single admixed population. To generate the plots, 100 chromosomes were sampled from each population at exponentially growing time points, and the WT centers were calculated for each chromosome in each sampled generation.

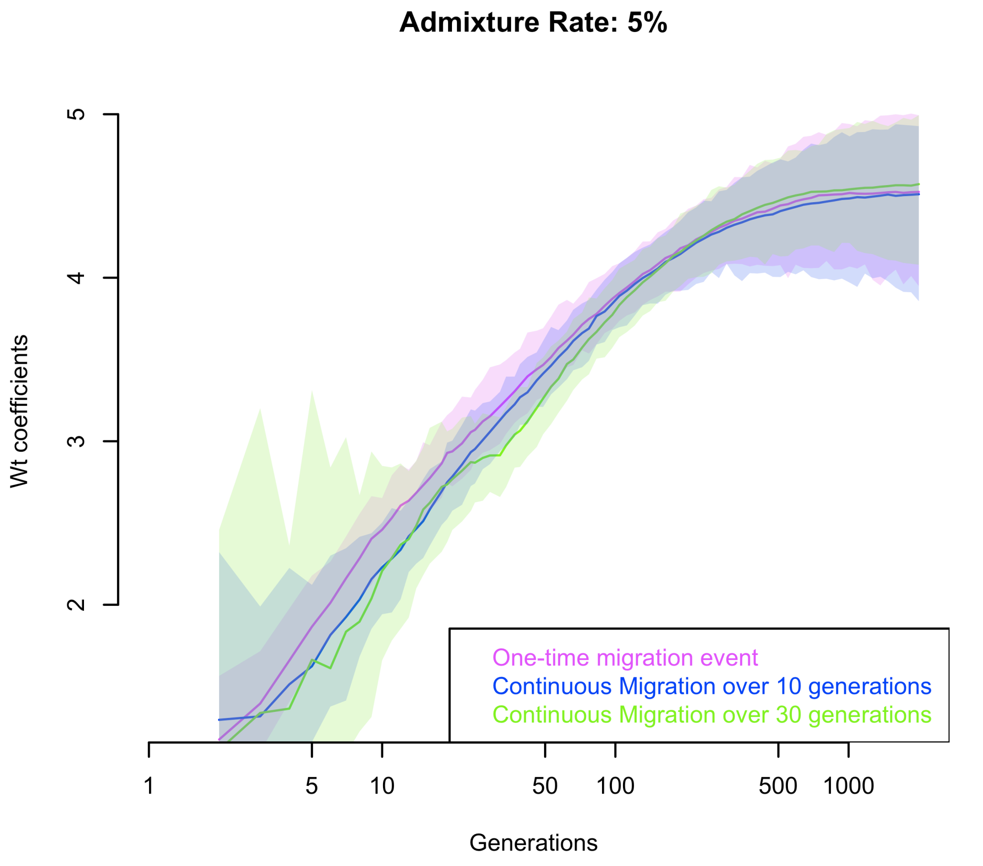

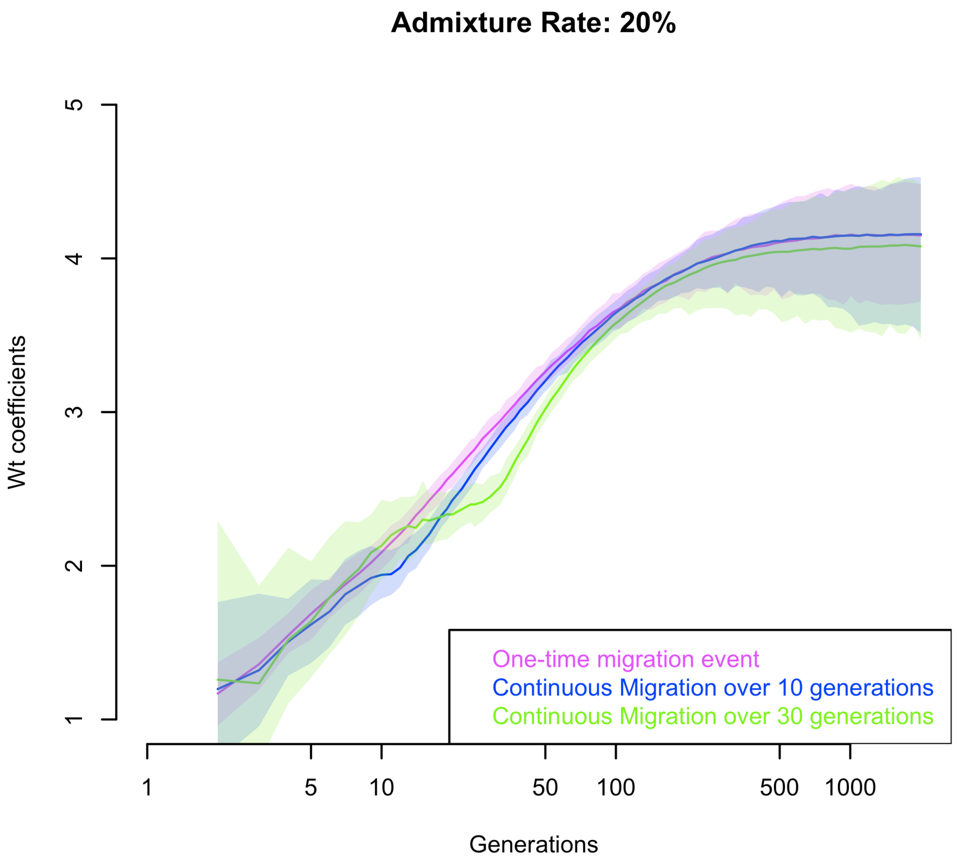

Figure S2. – Sensitivity of the StepPCO method with respect to continuous migration. Shown data are from 100 simulations for a one-time migration event and for continuous migration over a period of either 10 or 30 generations, and the migration rates of either 5% or 20%. 100 chromosomes were sampled from each simulated population at exponentially growing time points, the WT centers were calculated for each chromosome in each sampled generation. The mean WT centers and 95% CI for each simulated scenario are plotted as a function of time since admixture.

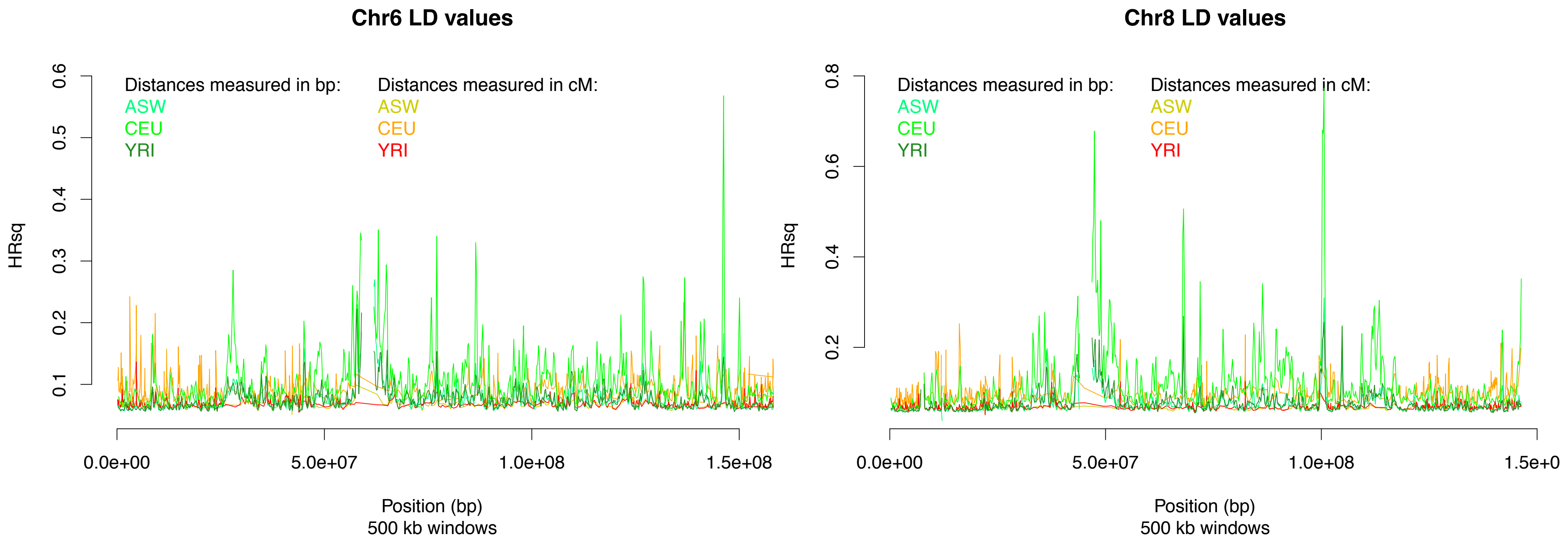

Figure S3. – Sensitivity of the StepPCO method with respect to levels of Linkage Disequilibrium. LD was calculated in fixed windows across chromosomes 6 and 8 in CEU, YRI and ASW. The size of the fixed window was chosen as a fraction of a chromosome length to correspond on average to either 500kb or 0.5cM.

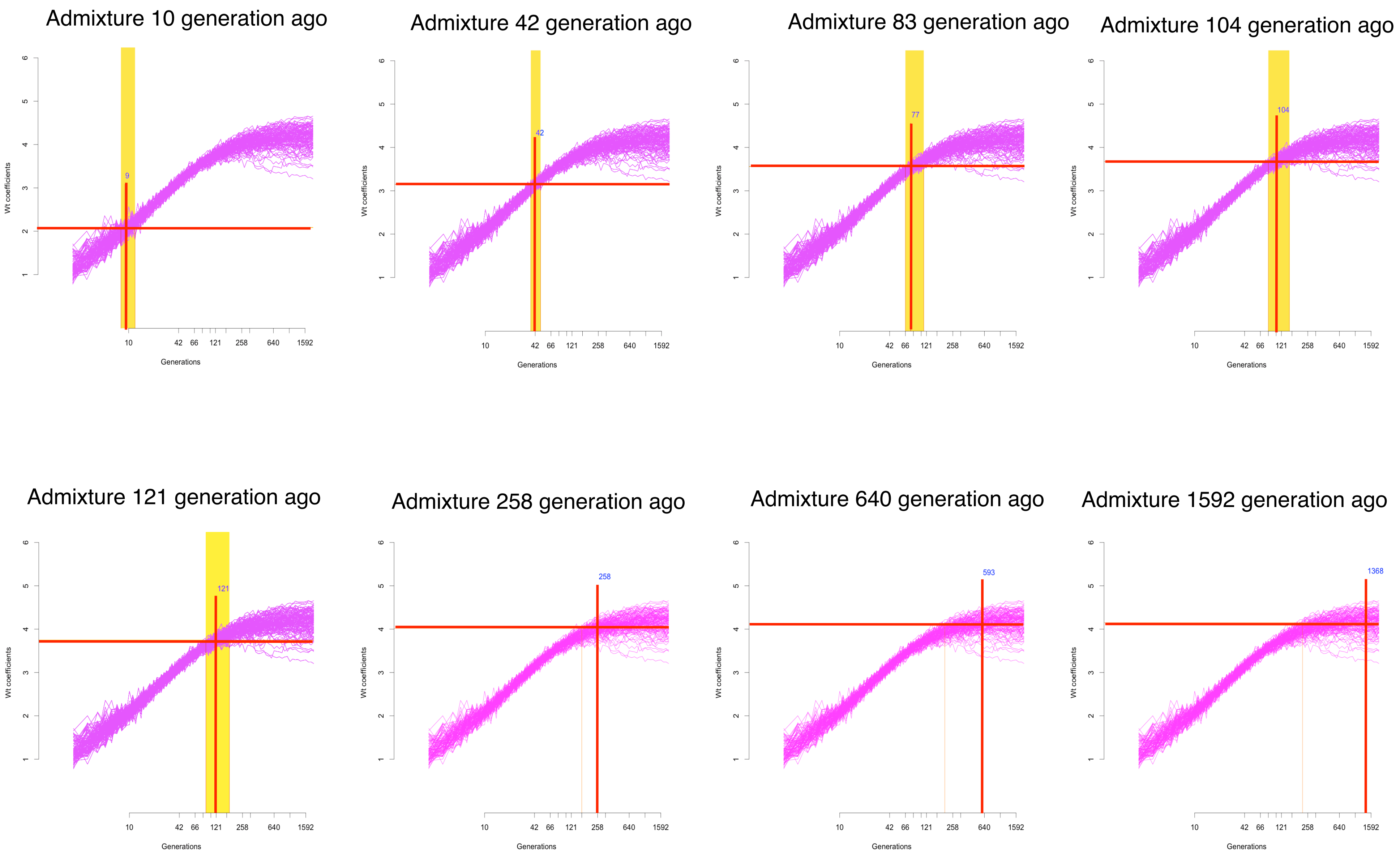

Figure S4. – Power analysis, indicating the admixture time estimated from simulated data consisting of 10 individuals, based on 100 simulations and a 20% migration rate for each time point. Each curve represents a single admixed population. To generate the plots, for each population 100 chromosomes were sampled and average WT centers calculated at exponentially growing time points . In addition, 10 individuals were sampled at random from one randomly chosen simulated population at 12 different time points (results for 8 time points are shown). Red horizontal lines indicate the WT centers calculated based on this sample of 10 individuals, vertical red lines indicate the inferred time since admixture, and the shaded box defines the confidence range.

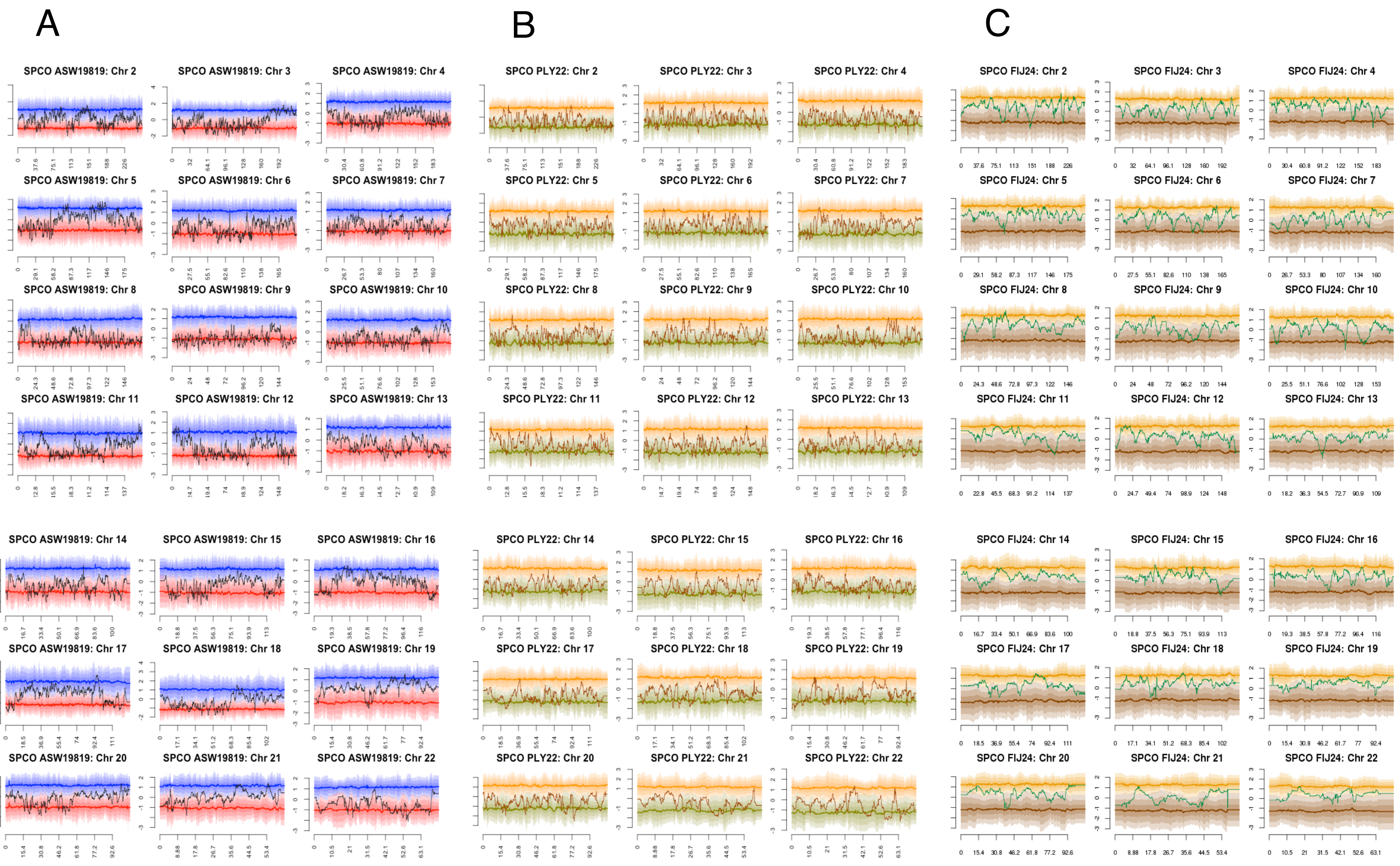

Figure S5. – Ancestry of one representative admixed individual estimated for each window across the genome using the StepPCO method. Solid lines centered around +1 and -1 indicate mean PC1 coordinates for each parental population; progressively lighter shading surrounding the mean indicate +/-1, +/- 2 or +/- 3 standard deviations from the mean. A) Genomewide ancestry estimates for the African-American individual from Figure 4 (where results for chromosome 1 are given); European (blue) and Yoruba (red) populations are used as parental groups. B) Genomewide ancestry estimates for the Polynesian individual from Figure 4 (where results from chromosome 1 are given); Borneo (green) and New Guinean (orange) populations are used as parental groups. C) Genomewide ancestry estimates for the Fijian individual from Figure 4 (where results from chromosome 1 are given); Polynesia (brown) and New Guinean (orange) populations are used as parental groups.

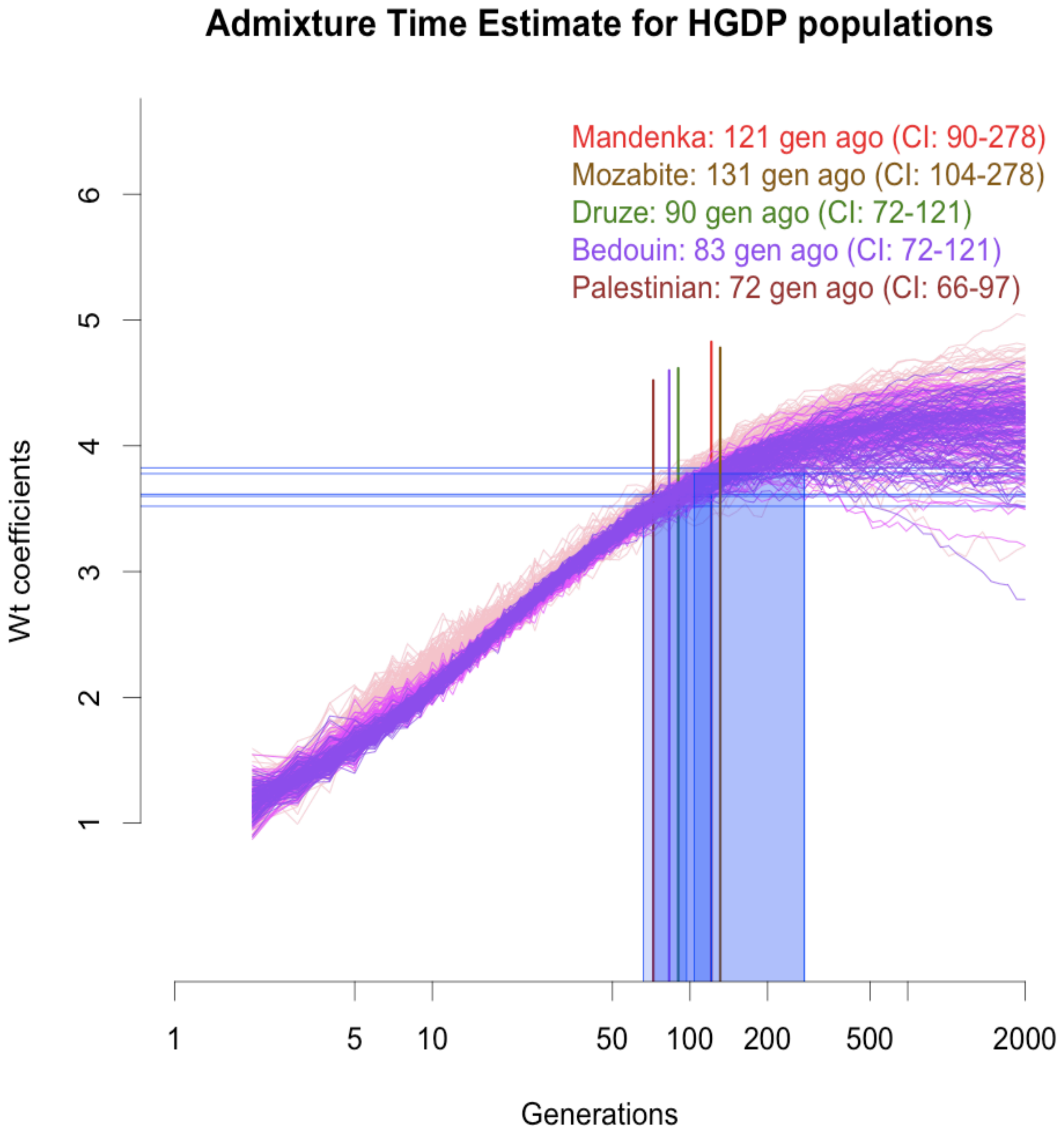

Figure S6. – Admixture time estimates for Mandenka, Mozabite, Druze, Bedouin and Palestinian. Red vertical lines indicate the time estimate, and shaded boxes define the confidence intervals.

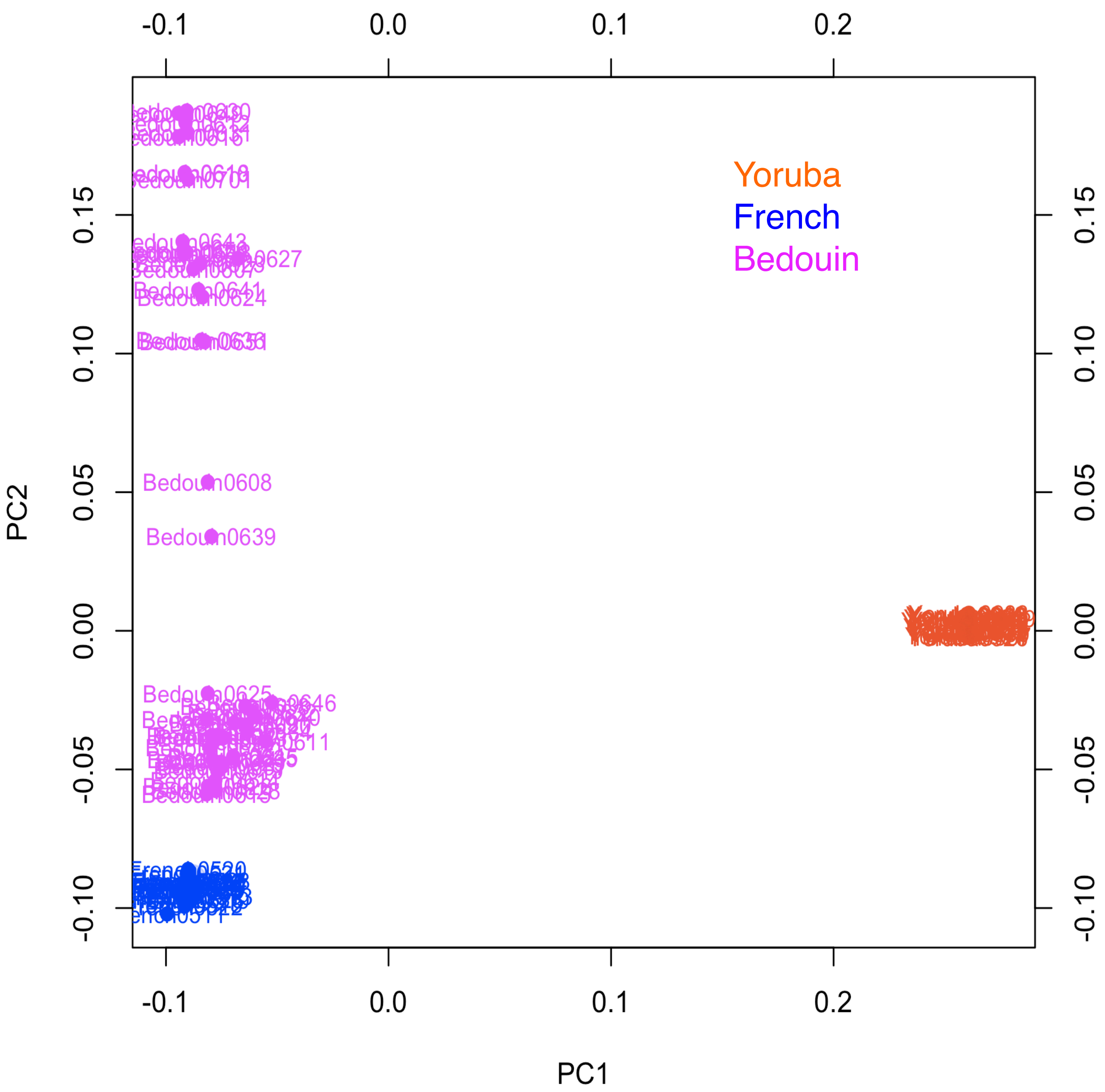

Figure S7. – PCA on genomewide data from French, Yoruba and Bedouin populations.

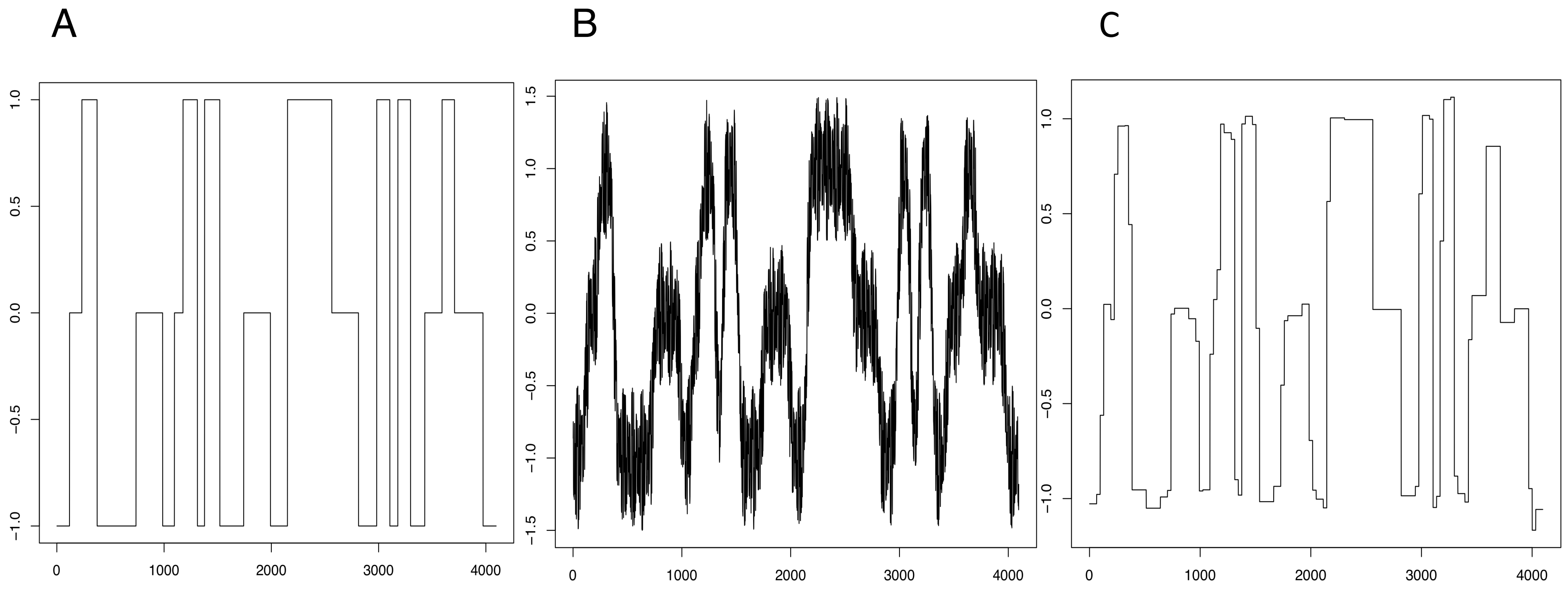

Figure S8. – An example of the noise reduction procedure using wavelet transform. Given a signal containing noise, one finds its wavelet coefficients, removes (sets to zero) coefficients describing high frequency, low amplitude oscillations, and uses an inverse wavelet transform to recover the signal without the noise. A) Hypothetical original signal. B) Same signal containing artificially-created noise. C) Recovered signal after wavelet transform.
